# ^18^F-Labelled PSMA-1007 shows similarity in structure, biodistribution and tumour uptake to the theragnostic compound PSMA-617

**DOI:** 10.1007/s00259-016-3447-9

**Published:** 2016-06-25

**Authors:** Frederik L. Giesel, Jens Cardinale, Martin Schäfer, Oliver Neels, Martina Benešová, Walter Mier, Uwe Haberkorn, Klaus Kopka, Clemens Kratochwil

**Affiliations:** 1 Department for Nuclear Medicine, University Hospital Heidelberg, INF 400, 69120 Heidelberg, Germany; 2 Division of Radiopharmaceutical Chemistry, German Cancer Research Center (DKFZ), Heidelberg, Germany


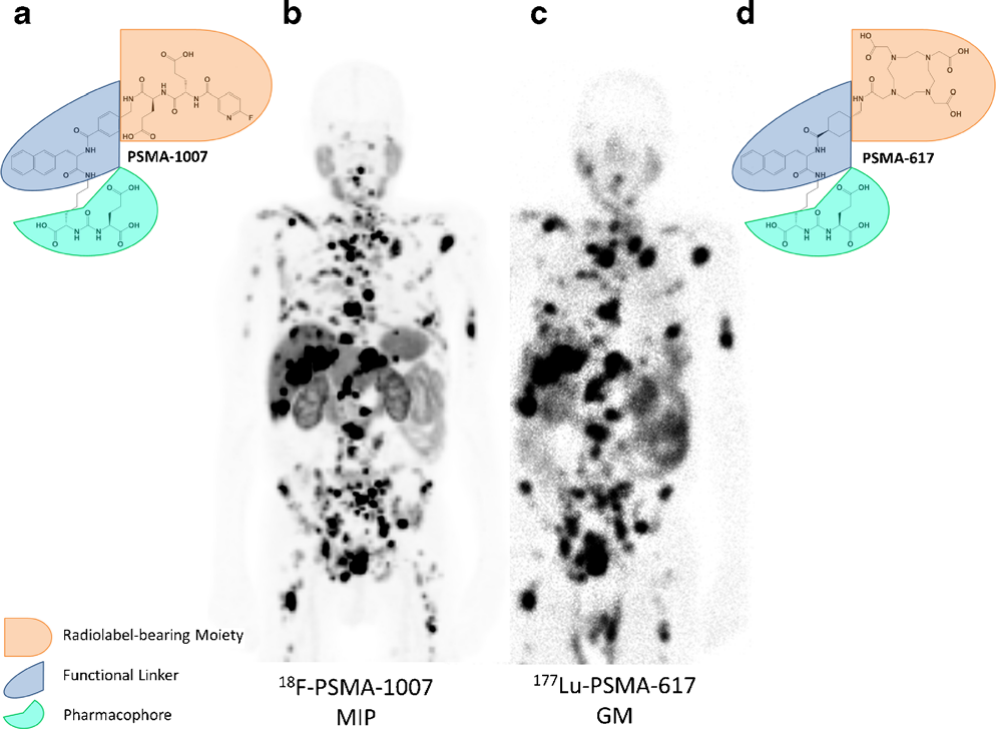


The biochemical and radiological responses to radionuclide therapy with ^177^Lu-PSMA-617 targeting prostate-specific membrane antigen (PSMA) make it a promising approach to the treatment of patients with metastatic castration-resistant prostate cancer (mCRPC) [1]. However, PSMA-617 has been reported to have slower tumour accumulation and clearance kinetics than PSMA-11, and the latter is still therefore the preferred diagnostic agent when labelled with generator-produced ^68^Ga which has a short half-life (68 min) [2]. A PSMA-targeting ^18^F-labelled PET tracer could be produced with higher activity in a cyclotron and the half-life (110 min) would allow both late imaging beyond 1 h after injection and shipping to satellite institutions. However, the structure of the currently most-used ^18^F-labelled PSMA tracer, ^18^F-DCFPyl, is different from that of PSMA-617, and like PSMA-11 it might be a suboptimal surrogate for stratifying patients according to their suitability for therapy with ^177^Lu-PSMA-617 [3].

Based on the scaffold of PSMA-617, the novel compound ^18^F-PSMA-1007 was developed. As shown in the image (**a**, **d**), PSMA-1007 shares the Glu-urea-Lys motif targeting the catalytic domain of PSMA and the naphthalene-based linker region considered to cotarget the hydrophobic accessory pocket [4], while in the radiolabel-bearing moiety glutamic acids were added to mimic the carboxylic acid groups of the DOTA chelator to retain the polar charge influencing clearance kinetics.

The image also shows a patient with mCRPC who was staged using ^18^F-PSMA-1007 (**b** PET 1 h after injection, maximum intensity projection) and treatment with ^177^Lu-PSMA-617 (**c** planar scan 24 h after injection, geometric mean). In analogy to the chemical structure, the uptake in tumour and normal organs is very similar with the two compounds.

Thus, ^18^F-PSMA-1007 and ^177^Lu-PSMA-617 seem to be a perfect theragnostic tandem. Due to the preferred physical characteristics of ^18^F for PET imaging and the possibility for large-scale production in a cyclotron, ^18^F-PSMA-1007 is also a promising alternative to ^68^Ga-PSMA-11 for diagnostic purposes. However, non-inferior diagnostic accuracy has still to be proven in a larger cohort.

## References

[1] Kratochwil C, Giesel FL, Stefanova M, Benešová M, Bronzel M, Afshar-Oromieh A (2016). PSMA-targeted radionuclide therapy of metastatic castration-resistant prostate cancer with Lu-177 labeled PSMA-617. J Nucl Med.

[2] Afshar-Oromieh A, Hetzheim H, Kratochwil C, Benesova M, Eder M, Neels OC (2015). The theranostic PSMA ligand PSMA-617 in the diagnosis of prostate cancer by PET/CT: biodistribution in humans, radiation dosimetry, and first evaluation of tumor lesions. J Nucl Med.

[3] Szabo Z, Mena E, Rowe SP, Plyku D, Nidal R, Eisenberger MA (2015). Initial evaluation of [(18)F]DCFPyL for prostate-specific membrane antigen (PSMA)-targeted PET imaging of prostate cancer. Mol Imaging Biol.

[4] Barinka C, Byun Y, Dusich CL, Banerjee SR, Chen Y, Castanares M (2008). Interactions between human glutamate carboxypeptidase II and urea-based inhibitors: structural characterization. J Med Chem.

